# SL-Miner: a web server for mining evidence and prioritization of cancer-specific synthetic lethality

**DOI:** 10.1093/bioinformatics/btae016

**Published:** 2024-01-19

**Authors:** Xin Liu, Jieni Hu, Jie Zheng

**Affiliations:** School of Information Science and Technology, ShanghaiTech University, Shanghai 201210, China; School of Life Science and Technology, ShanghaiTech University, Shanghai 201210, China; School of Information Science and Technology, ShanghaiTech University, Shanghai 201210, China; Shanghai Engineering Research Center of Intelligent Vision and Imaging, Shanghai 201210, China

## Abstract

**Summary:**

Synthetic lethality (SL) refers to a type of genetic interaction in which the simultaneous inactivation of two genes leads to cell death, while the inactivation of a single gene does not affect cell viability. It significantly expands the range of potential therapeutic targets for anti-cancer treatments. SL interactions are primarily identified through experimental screening and computational prediction. Although various computational methods have been proposed, they tend to ignore providing evidence to support their predictions of SL. Besides, they are rarely user-friendly for biologists who likely have limited programming skills. Moreover, the genetic context specificity of SL interactions is often not taken into consideration. Here, we introduce a web server called SL-Miner, which is designed to mine the evidence of SL relationships between a primary gene and a few candidate SL partner genes in a specific type of cancer, and to prioritize these candidate genes by integrating various types of evidence. For intuitive data visualization, SL-Miner provides a range of charts (e.g. volcano plot and box plot) to help users get insights from the data.

**Availability and implementation:**

SL-Miner is available at https://slminer.sist.shanghaitech.edu.cn.

## 1 Introduction

Synthetic lethality (SL) refers to a type of genetic interaction in which the simultaneous inactivation of two genes results in cell death, while the inactivation of a single gene does not affect cell viability. This phenomenon was first described by Calvin Bridges in 1922 who observed that specific combinations of mutated genes in the model organism *Drosophila melanogaster* had lethal effects (Nijman 2011). These combinations of genes were defined as synthetic lethal gene pairs. Given that gene mutations differentiate cancer cells from normal cells, Hartwell *et al.* first proposed the strategy of using synthetic lethality as anti-cancer drug targets (Hartwell *et al.* 1997). For example, the loss-of-function of a tumor suppressor gene in cancer cells renders the gene undruggable, but its SL partner gene, if existing, can still be a selective drug target. As such, the SL-based strategy allows indirect targeting of undruggable cancer mutations and thereby expands the space of anti-cancer treatment targets.

Currently, there are mainly two types of approaches to identifying SL interactions: experimental screening and computational prediction. Due to the vast search space of potential gene pairs, experimental screens are often time-consuming and costly. As an alternative, computational methods such as DAISY (Jerby-Arnon *et al.* 2014), SL^2^MF (Liu *et al.* 2020), KG4SL (Wang *et al.* 2021), and NSF4SL (Wang *et al.* 2022b) can make large-scale SL predictions. The computational methods mainly include two types: machine learning-based methods and hypothesis-based statistical inference methods.

The machine learning-based methods capture the associations between input features of genes and SL relationships by supervised learning from known SL labels. For example, DiscoverSL (Das *et al.* 2019) selects gene features from multi-omics data (i.e. mutation, gene expression and copy number alteration data from TCGA) and then predicts SL interactions using random forest (RF). GCATSL (Long *et al.* 2021) is a graph contextual attention network model integrating various biological data to construct feature graphs, and a hierarchical attention mechanism is designed to learn feature-specific gene representations for predicting SL pairs. PT-GNN (Long *et al.* 2022) uses different sources of biological data to learn their features in a pre-training GNN framework. The model can be used for link prediction tasks including SL prediction and DTI (drug–target interaction) prediction. PiLSL (Liu *et al.* 2022) extracts representations of gene pairs from a knowledge graph and integrates them with gene features from multi-omics data to predict SL gene pairs. While these methods can detect SL interactions effectively, their output often lacks clear evidence supporting their predictions. Providing SL evidence could help biologists understand potential mechanisms behind SL interactions and accelerate functional and clinical studies of the predicted novel SLs.

Hypothesis-based statistical inference methods rely on biological assumptions derived from the concept of SL and patterns shared by existing SL interactions. For instance, some assumptions are that SL genes are frequently co-expressed, share similar functions, or exhibit mutual exclusivity. Statistical patterns satisfying these assumptions can be used to infer SL interactions. For example, SynLeGG (Kim *et al.* 2017) uses mutually exclusive loss signatures to discover SL interactions. However, methods relying solely on a single assumption may introduce bias and lead to many false positive SLs. Integrating inferred results based on diverse assumptions may help reduce the false positive rate. For example, the robust rank aggregation (RRA) method (Kolde *et al.* 2012) produces fewer false positives in finding essential gene regulators by integrating various assumptions (Song *et al.* 2021).

Although some SL interactions are conserved across various human cancers, many of them are only observed in a few specific cancers (Ryan *et al.* 2018). A study based on CRISPR-Cas9 screens identified SL gene pairs in each of three cell lines and found that only approximately 10% of these SL interactions were shared by two cell lines, and that no SL gene pair is identified in all the three cell lines (Shen *et al.* 2017). Despite the emerging popularity of computational methods for predicting SLs, most of them were designed for pan-cancer SLs without considering the cancer context specificity.

Furthermore, these computational methods are often deployed in the form of source code, such as Python or R code. It is challenging for biologists with limited programming skills to handle raw data and run the source codes to discover novel SL interactions. Although some user-friendly web servers have been developed for predicting SL, they exhibited some limitations. For example, SL-BioDP (Deng *et al.* 2019) is a comprehensive web tool based on machine learning methods for predicting SL interactions, which provides visualization and analysis of results. Although it harnesses multi-type sources as the features of the machine learning models, it is trained on limited SL label data. G2G (Almozlino *et al.* 2020) is a web server that employs an improved random forest (RF) algorithm to predict SL interactions, which provides one page to input the querying genes and output the predicted results with tables and graphs. However, it does not provide SL evidence to assist understanding the prediction results.

To address these issues, we developed SL-Miner, a user-friendly web server designed to mine SL evidence and prioritize gene pairs for specific cancer types. It employs multiple statistical methods for mining SL evidence between a primary gene and a few partner gene candidates for a given cancer type. The web server employs the aforementioned RRA algorithm to prioritize the candidate SL partner genes. It is noteworthy that the prioritization is an integration analysis of the mined SL evidence, rather than direct SL prediction. The partner gene candidates can be obtained in various ways, such as the genes that are interesting to users, genes involved in some pathways in KEGG (Kanehisa and Goto 2000) or SL partner genes predicted by machine learning-based methods. For these query gene pairs, SL-Miner would then offer SL evidence by statistical inference and analysis of various types of data. To visualize the SL evidence, SL-Miner would draw different types of statistical charts, including volcano plot, box plot and scatter plot. The web server incorporates the preprocessed high-throughput genetic and chemogenetic screening data, as well as multi-omics data from cancer cell lines in DepMap (https://depmap.org/portal) and patient-level clinical studies in TCGA (Weinstein *et al.* 2013).

## 2 Functionality

### 2.1 Overview of SL-Miner core functions

**Figure 1.**
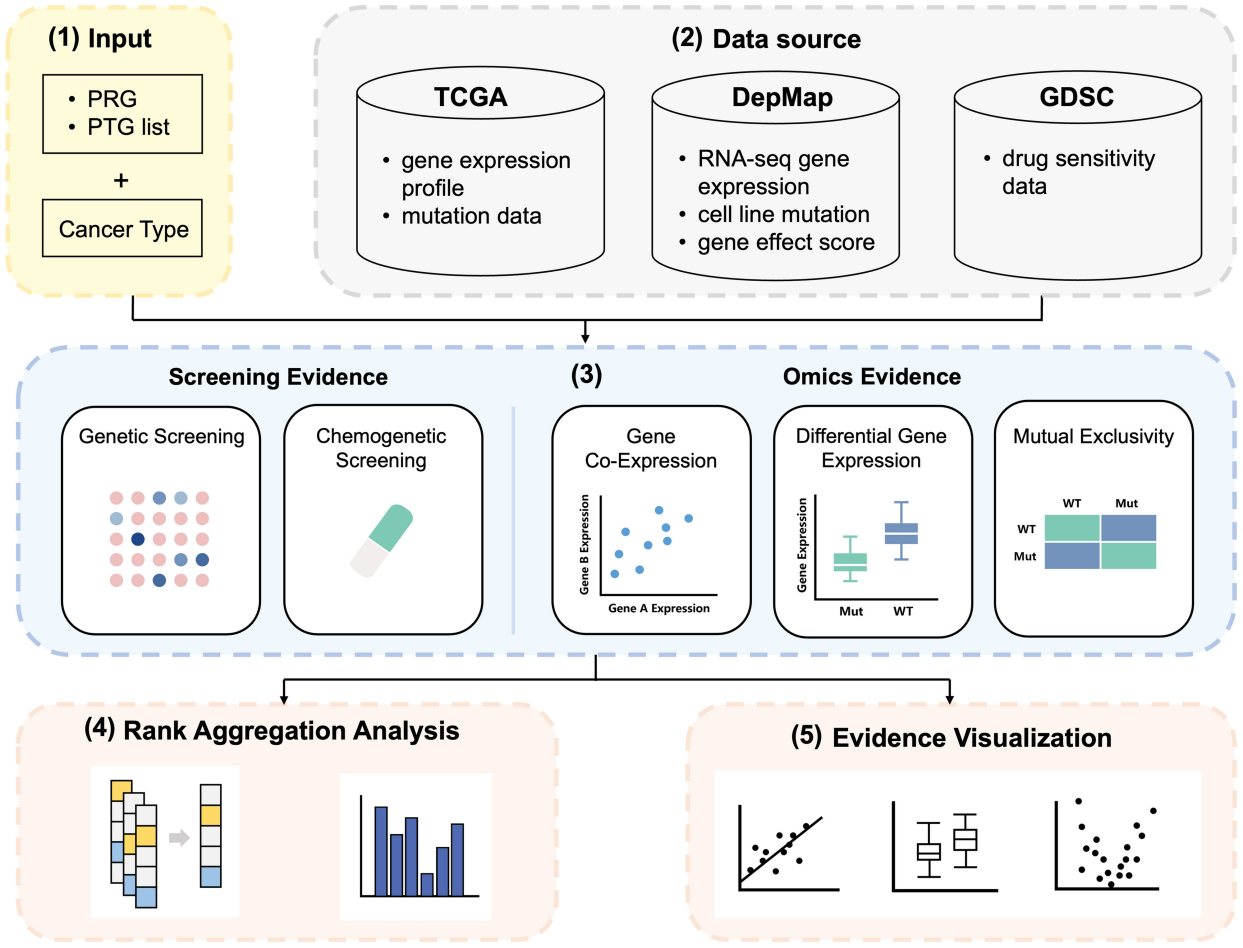
Pipeline of SL-Miner. (1) Input: a primary gene (PRG) of interest to the user, a list of candidate partner genes (PTGs) and a cancer type. (2) Built-in data sources: clinical patients, cancer cell lines and high-throughput data from TCGA, DepMap and GDSC, respectively. (3) SL Evidence Mining Modules: SL evidence types can be categorized into screening evidence and omics evidence. Screening evidence is derived from high-throughput screens based on gene perturbations (CRISPR and drug). Omics evidence is obtained from the concept of SL and omics data. (4) Rank Aggregation Analysis: Based on the ranking results from the SL evidence mining, rank aggregation analysis is performed to achieve a robust ranking of candidate partner genes. (5) Evidence visualization: The SL evidence is visualized in various kinds of charts (e.g. volcano plot, box plot, scatter plot), which can be downloaded directly from SL-Miner.

The objective of SL-Miner is to mine various types of evidence for cancer-specific SL relationships between a primary gene and a list of SL partner gene candidates and then prioritize the potential SL partner genes based on the mined evidence. Specifically, the SL-Miner web server consists of three core functional modules (see Fig. 1). Two of these modules, namely Screening Evidence module and Omics Evidence module, are designed for statistical testing. The Screening Evidence module determines whether the viability effect score of gene A or IC50 of a drug targeting gene A in a cancer with mutation of gene B is significantly lower than that in the same cancer without mutation of gene B. The viability effect scores, which are derived from high-throughput CRISPR screens (DepMap), denote the extent to which cell growth is inhibited following gene knockout. The IC50, which is obtained from high-throughput drug screens (GDSC) (Yang *et al.* 2013), represents the concentration of a drug required to inhibit cell viability by 50%. The Omics Evidence module assesses whether a pair of genes may share biological mechanisms with existing SLs based on gene expression and mutation data (DepMap and TCGA). The third module uses the RRA algorithm to integrate various types of SL evidence and rank the partner gene candidates. For a detailed description of these modules, please refer to Supplementary Information.

### 2.2 Input and output description

To begin with, users provide a primary gene and candidate SL partner genes as input. The candidate partner genes can be obtained in three ways: (i) manually entering a list of candidate genes of interest (including genes predicted by ML-based methods); (ii) selecting a pre-defined gene set collected from the Molecular Signatures Database (MSigDB) (Liberzon *et al.* 2011); (iii) retrieving SL pairs from SynLethDB (a comprehensive database that collects SL gene pairs from multiple data sources) (Wang *et al.* 2022a). Once users successfully create a list of candidate partner genes, it can be used in every module throughout the entire analysis process.

In the evidence mining modules, a TCGA or DepMap cancer type should be selected to focus on a specific context. The cancer types are ranked according to the mutation rate of the primary gene. If users are interested in multiple cancer types with high mutation frequencies for a primary gene (usually not a large number of cancer types), they can manually change the Cancer Type on the web-server and obtain analysis results for these specified cancer types. For detailed descriptions of the input and output of each module, please refer to Supplementary Information.

## 3 Case study

Users can define their interested candidate partner gene lists as the input to SL-Miner. Our case study shows two examples of either using genes participating in the same pathway with the primary gene, or using paralogous genes of the primary gene as the partner candidates, to mine SL evidence and prioritize the potential SL interactions.

### 3.1 SL interactions between BRCA1 and DNA damage repair-related genes

BRCA1 plays a crucial role in repairing double-strand breaks (DSBs) of DNA by Homologous Recombination Repair (HRR) (Prakash *et al.* 2015). Defects in BRCA1 are significantly correlated with the risk of breast cancer. In recent years, several genes such as PARP1/2, ATR, CHK1, and WEE1 etc. (Cleary *et al.* 2020), which also participate in DNA damage repair (DDR), have been suggested to have SL interactions with BRCA1 and studied in clinical trials. However, still more SL partners of BRCA1 remain to be discovered. Genes participating in DDR pathways are potential candidates. In this case study, we use SL-Miner to prioritize potential SL interactions between BRCA1 and DDR-related genes in breast cancer. To this end, we constructed a list of DDR-related genes from the MSigDB database, which contains tens of thousands of annotated gene sets.

In the following, we shall first get the aggregated ranking results of the selected DDR-related partner genes and narrow down to those top candidates. Then, we will focus on the most interesting candidates, such as those candidates not reported, and mine specific evidence supporting their SL interactions with BRCA1. The Prioritization module outputs an aggregated ranking of the partner genes based on the evidence testing. Among the top 10 candidates, 6 partner genes have not yet been recorded in SynLethDB (i.e. MBD4, POLE3, TOPBP1, RAD51D, POLQ, and PPP5C), which could be potential novel SL partners of BRCA1. Clues to these novel SL interactions can be found in the evidence testing modules. Here we take the SL interaction between BRCA1 and TOPBP1, a scaffold protein involved in G2-M checkpoint signaling in DDR (Laine *et al.* 2021), as an example. In the Gene Co-Expression test, TOPBP1 is significantly co-expressed with BRCA1. On the TCGA dataset, the Pearson correlation is 0.504 and the non-correlation test *P*-value is 8.51e^−80^. On the DepMap dataset, the Pearson correlation is 0.704, and the *P*-value is 1.73e^−10^. In the Genetic Screening and Differential Gene Expression tests, although the testing results show no significance (*P*-value > 0.05), the test *P*-value of TOPBP1 surpasses most of the other partner genes in the aggregated ranking. The test effect size of the Genetic Screening testing shows that TOPBP1’s viability effect score is lower in BRCA1 deficient cell lines, indicating that in the case of BRCA1 mutation TOPBP1 tends to be more essential. Also, according to the result of Differential Gene Expression testing, TOPBP1 tends to have higher expression levels in BRCA1-deficient cell lines.

We conducted a literature survey and found that there is a significant correlation between the BRCA1 protein expression and TOPBP1 protein expression among Non-Small Cell Lung Cancer (NSCLC) patients. In addition, the negative expression of both proteins shows a positive impact on the prognosis and survival of the NSCLC patients (Wang *et al.* 2015). These findings are consistent with the results of our case study using SL-Miner. Overall, TOPBP1 is a promising and novel SL partner of BRCA1, but more experimental results are still needed to verify this predicted SL relationship.

### 3.2 SL interactions between paralogous gene pairs

Paralogous genes arise from gene duplications, which often share common functions to compensate for each other’s loss. Pairs of paralogs are significantly more likely to be synthetic lethal than random gene pairs (Ryan *et al.* 2023). Focusing on the relatively small subset of paralogs is a potential way of discovering new SL interactions. In this case study, we use SL-Miner to prioritize potential SL interactions between paralogous gene pairs. We obtained primary genes from the top-ranking paralogous SL pairs from three independent combinatorial screenings (Dede *et al.* 2020, Parrish *et al.* 2021, Thompson *et al.* 2021) and prioritized their SL partners within their paralogues.

As an example, we focus on the SL interaction between CCNE1 and CCNE2 in Colorectal cancer. CCNE1 and CCNE2 are both Cyclin E genes, which associate with CDK2 to control cell cycle progression and DNA replication. Previous research suggested that expression of CCNE1 and CCNE2 could synergistically affect the overall survival in hepatocellular carcinoma (HCC) patients and HCC progression requires the expression of any E-cyclin (Sonntag *et al.* 2018). Simultaneously downregulating CCNE1 and CCNE2 inhibits NSCLC progression (Liang *et al.* 2020). We set CCNE2 as the primary gene and observed that CCNE1 ranked in the 4th place out of all 18 CCNE1’s paralogues, in consistency with the above results. A more comprehensive illustration of the analysis process and interpretation of the results can be found in Supplementary Information.

## 4 Conclusion

SL is a promising strategy for cancer treatment. We have developed a web server named SL-Miner to prioritize candidate SL partner genes for a primary gene within specific contexts by mining and integrating various types of evidence. The context-specific SL evidence can provide biological insights into the genetic vulnerabilities of cancer cells which can help develop personalized cancer therapies. Integrating the mined SL evidence into a ranking list can reduce the false positive rate and improve the reliability of SL prediction. Additionally, SL-Miner provides figures and tables that can be downloaded and used for publication. It can be accessed at https://slminer.sist.shanghaitech.edu.cn.

## Supplementary Material

btae016_Supplementary_Data
