# Supplementary material for: SL-Miner: a web server for mining evidence and prioritization of cancer-specific synthetic lethality
Source: Bioinformatics. 2024 Jan 19;40(2):btae016. doi: 10.1093/bioinformatics/btae016 (PMC10868331; doi:10.1093/bioinformatics/btae016)
Supplement: btae016_Supplementary_Data [file btae016_supplementary_data.pdf]

Supplementary Information
for
SL-Miner: a web server for mining evidence and prioritization of cancer-specific synthetic lethality

Xin Liu[1], Jieni Hu[2] and Jie Zheng[1,3,*]

[1] School of Information Science and Technology, ShanghaiTech University, Shanghai, 201210, China,

[2] School of Life Science and Technology, ShanghaiTech University, Shanghai, 201210, China,

[3] Shanghai Engineering Research Center of Intelligent Vision and Imaging, Shanghai, 201210, China.

[*] To whom correspondence should be addressed.

Contents

1 Web server Information **3**

1.1 Implementation 3

1.2 Input and output 3

2 Methods and Materials **5**

2.1 Data collection and pre-processing 5

2.1.1 Clinical patient data 5

2.1.2 Cancer cell line data 5

2.1.3 Genetic screening data 5

2.1.4 Chemogenetic screening data 5

2.1.5 Pre-defined gene sets 6

2.2 Synthetic lethality evidence mining 6

2.2.1 Screening evidence 6

2.2.2 Omics evidence 6

2.3 Rank aggregation analysis 7

3 Evaluation **7**

3.1 Golden standard dataset 7

3.2 Evaluation of robust rank aggregation performance 7

4 Case Studies **8**

4.1 SL interactions between BRCA1 and DDR-related genes 9

4.2 SL interactions between paralog gene pairs 11

5 References **14**

1 Web server Information

SL-Miner is a user-friendly web server designed to mine evidence of synthetic lethality (SL) and prioritize SL gene pairs for specific cancer types. It employs multiple statistical methods for mining SL evidence between a primary gene and a few partner gene candidates for a given cancer type. The evidence can give clues to potential SL interactions in terms of various statistical tests, and it can also be used to prioritize the candidate SLs. In terms of application, SL-Miner can be employed in conjunction with SL prediction methods and existing SL databases. For SLs predicted by other methods or retrieved from SL databases, SL-Miner can provide evidence through statistical inference and analysis of various types of data to rank a list of candidate SLs. To visualize the SL evidence, SL-Miner can draw various types of statistical charts, including volcano plot, box plot and scatter plot.

1.1 Implementation

The SL-Miner web server employs the Representational State Transfer (REST) design style to separate the front-end and back-end architecture. The front end is responsible for a user-friendly interface display, and the back end handles application logic. The React.js (https://react.dev/) web user interface (UI) framework is used for the front-end development, with most webpages created using the Element UI library (https://element.eleme.io/). Additionally, the ECharts.js (https://echarts.apache.org/) and D3.js (https://d3js.org/) libraries are employed to generate figures or tables for displaying results. On the back end, the web application programming interface (API) is developed by Flask (https://flask.palletsprojects.com/), and data resources are stored in the non-relational database MongoDB (https://www.mongodb.com/). Lastly, Nginx (https://nginx.org/) is selected as the reverse proxy agent.

1.2 Input and output

Genetic Screening. Users are required to provide a primary gene, a set of partner gene candidates, and a specific cancer type as input. This module returns p-values from one-sided Wilcoxon rank sum tests performed on the viability effect scores of partner gene candidates between the Mutation (Mut) Group (cell lines with mutations of the primary gene) and Wild Type (WT) Group (cell lines without primary gene mutation). The results can be sorted by the p-values. Visual representations include a volcano plot displaying the $-\log_{10}$(P-value) and viability effect sizes of all combinations between the primary gene and partner gene candidates, and a box plot showing the distribution of viability effect scores of an individual partner gene from cell lines grouped by the Mut/WT status of the primary gene.

Chemogenetic Screening. Users are required to provide a primary gene and a drug targeting one of the SL partner gene candidates. This module returns p-values from one-sided Wilcoxon

rank sum tests performed on the IC50 values of the drug targeting candidate SL partner genes between the Mut group and WT group among various cancer types. Visual representations include a volcano plot displaying the $-\log_{10}$(P-value) and IC50 effect sizes of all combinations between the primary gene and SL partner gene candidates, and a box plot showing the distribution of the IC50 effects of the drug grouped by primary gene Mut/WT status under a specific cancer type.

Gene Co-Expression. Users are required to provide a primary gene, a set of SL partner gene candidates, and a specific cancer type from TCGA (https://portal.gdc.cancer.gov/) or DepMap (https://depmap.org/portal/). This module returns the results derived from a Pearson correlation test conducted on gene expression of the primary gene and the partner gene candidates. Visual representations include a volcano plot displaying the $-\log_{10}$(P-value) and correlation coefficients of all combinations between the primary gene and partner gene candidates, and a scatter plot showing the gene expression correlation between an individual partner gene and the primary gene.

Differential GeneExp. Users are required to provide a primary gene, a set of SL partner gene candidates, and a specific cancer type from TCGA or DepMap. This module returns a p-value derived from one-sided Wilcoxon rank sum tests performed between the expression levels of the partner gene candidates from the Mut group and the WT group respectively. Visual representations include a volcano plot displaying the $-\log_{10}$(P-value) and expression effect sizes of all combinations between the primary gene and partner gene candidates, and a box plot showing the distribution of the expression levels of an individual partner gene from patients (TCGA) or cell lines (DepMap) grouped by the Mut/WT status of primary gene.

Mutual Exclusivity. Users are required to provide a primary gene, a set of SL partner gene candidates, and a specific cancer type. This module returns the results of one-sided Fisher's exact tests in a tabular form, including the 2×2 contingency tables, the $\log_2$ values of the odds ratios, and the derived p-values.

Prioritization. Users are required to provide a primary gene, a set of SL partner gene candidates, and a specific cancer type from TCGA and DepMap. Integrated results of the algorithm of robust rank aggregation (RRA) will be returned in a tabular form, including RRA scores and RRA ranks. A bar plot will be generated to show the rank of each candidate SL partner gene with respect to each individual evidence test in the Screening Evidence (Genetic Screening and Chemogenetic Screening) and Omics Evidence (Gene Co-Expression, Differential GeneExp, and Mutual Exclusivity) modules.

2 Methods and Materials

2.1 Data collection and pre-processing

2.1.1 Clinical patient data

We first collected gene expression profiles and somatic mutation data from The Cancer Genome Atlas (TCGA) covering 33 different cancer types. The gene expression profiles were processed by $\log_2$-transformation of Fragments Per Kilobase of transcript per Million mapped reads (FPKM) values. The mutation data include mutant gene symbols and mutation types. Non-functional mutations, such as silent mutations (i.e. synonymous mutations) and mutations in intronic regions, were removed, while functional mutations (i.e. loss-of-function and gain-of-function) were retained. Samples with gene mutations (either loss-of-function or gain-of-function) were labeled as "Mutation Type" and samples without gene mutations were labeled as "Wild Type" Both types of omics data (i.e. gene expression and somatic mutation) were downloaded from the GDC Data Portal (https://portal.gdc.cancer.gov/) in June 2022.

2.1.2 Cancer cell line data

Gene expression and mutation data of cancer cell lines collected in the Cancer Cell Line Encyclopedia (CCLE) were downloaded from the DepMap consortium website (version DepMap22Q2, https://depmap.org/portal/download). The gene expression data of RNA-seq were $\log_2$(TPM+1) transformed. The cell line mutation data were encoded as a binary matrix (with rows representing genes and columns representing cell lines), where the value of an entry is 1 if the cell line contains at least one functional mutation (loss-of-function or gain-of-function) in the gene and 0 otherwise.

2.1.3 Genetic screening data

Genetic screening data were curated from the 2022Q2 DepMap release, containing the viability effect scores of 17,387 genes among 1,086 cell lines. Gene effect scores of cell viability were derived from CRISPR knockout screens published by Broad's Achilles (https://depmap.org/portal/) and Sanger's SCORE projects (https://score.depmap.sanger.ac.uk/downloads). It is worth noting that the effect scores were calculated from raw read count data through the Chronos pipeline (Dempster *et al.*, 2021).

2.1.4 Chemogenetic screening data

Drug sensitivity data of cancer cell lines were downloaded from the database of Genomics of Drug Sensitivity in Cancer (GDSC, https://www.cancerrxgene.org/). This chemogenetic screening dataset includes the drug response values of IC50 (i.e. the half-maximal inhibitory concentration) of 518 drugs on 988 cancer cell lines in the GDSC database.

2.1.5 Pre-defined gene sets

SL-Miner contains 186, 1,654, and 7,751 pre-defined gene sets derived from KEGG (Kanehisa and Goto, 2000), Reactome (https://reactome.org/), and GO (https://geneontology.org/), respectively. These gene sets can be used to initialize the SL partner gene candidates as input of SL-Miner. Additionally, SL-Miner includes 35,943 SL gene pairs from SynLethDB 2.0 (Wang *et al.*, 2022) (https://synlethdb.sist.shanghaitech.edu.cn/), which can be used to initialize the SL partner gene candidates.

2.2 Synthetic lethality evidence mining

SL evidence is divided into two categories: screening evidence and omics evidence. The screening evidence is from high-throughput gene perturbation screens (e.g. CRISPR screening and drug screening). The omics evidence is obtained from statistical analysis of omics data based on the concept of SL.

2.2.1 Screening evidence

Genetic Screening test. The genetic screening evidence test is to determine whether the viability effect scores of gene A under a specific cancer type with gene B mutation are significantly lower than that under the same cancer type without gene B mutation using a one-sided Wilcoxon rank sum test (Gallo *et al.*, 2022). The gene pairs that pass this test are considered to have SL interactions in terms of genetic screening evidence.

Chemogenetic Screening test. Similarly, the chemogenetic screening evidence test is to determine whether the IC50 values of a drug targeting gene A under a specific cancer type with gene B mutation are significantly lower than those under the same cancer type without gene B mutation using a one-sided Wilcoxon rank sum test (Zhang *et al.*, 2021). The gene pairs that pass the test are considered to have SL interactions in terms of chemogenetic screening evidence.

2.2.2 Omics evidence

In contrast to screening evidence, omics evidence mainly relies on multi-omics data (e.g. gene expression and somatic mutation). The omics evidence of SL is based on biological prior knowledge, such as the observations that SL genes are frequently co-expressed, share similar functions, or exhibit mutual exclusivity.

Co-Expression test. This test is based on the observation that genes in an SL pair are frequently co-expressed and may share similar biological functions (Jerby-Arnon *et al.*, 2014a). Correlation analysis was performed to test the correlation between the expression of two genes.

Diff-Expression test. This test is based on the observation that SL partners of a mutated gene (primary gene) tend to have higher expression in samples harboring the mutation (Sinha *et al.*, 2017). A rank sum test was used to test the differentiated gene expression of partner genes between cell lines with and without mutation of the primary gene.

Mutual Exclusivity test. This test aims to detect mutual exclusivity with respect to specific genetic events (Deng *et al.*, 2019). A Fisher's exact test was used to assess the significance of these genetic events.

2.3 Rank aggregation analysis

The output from each of the above SL evidence tests is in the form of gene ranking, based on which rank aggregation analysis is performed to achieve a robust ranking of candidate SL partner genes. An order statistics-based approach named robust rank aggregation (RRA) method (Kolde *et al.*, 2012) was used to conduct rank aggregation analysis. The output scores of this method was p-value, and we defined the p-value as the robust rank aggregation score (i.e. RRA score). The cut-off of RRA score is 0.05. Specifically, if an RRA score is less than 0.05, the result is considered to be significant which also indicates that the gene pair is likely an SL. The final ranking of candidate SL pairs is determined based on the RRA score, where a lower RRA score indicates a more concordant high ranking.

3 Evaluation

3.1 Golden standard dataset

To evaluate the performance of the prioritization module, we constructed a golden standard dataset based on CRISPR screening data that reveals highly confident SL interactions. The dataset is comprised of clinically relevant SLs (Parameswaran *et al.*, 2019), SL pairs derived from genetic interaction (GI) maps of 220K gene pairs in the K562 and Jurkat cell lines (Horlbeck *et al.*, 2018) (gene pairs with GI scores in both cell lines less than -3.0 were selected), SL pairs validated through combinatorial screening in six cell lines (Najm *et al.*, 2018) (gene pairs with FDR less than 0.05 were selected) and experimentally derived SLs in three cell lines (Shen *et al.*, 2017). We also labeled the cancer types of these SL interactions by using the provided cancer cell line information.

3.2 Evaluation of robust rank aggregation performance

For each SL pair from our golden standard dataset, a collection composed of its partner gene and other 199 randomly selected genes from all human genes was constructed (total 200 genes as input for candidate partner genes). Then we conducted the aforementioned robust rank

aggregation (RRA) analysis using the constructed list of partner genes as input. We observed the rank scores of the candidate partner genes. The result shows that the partner genes according to the golden standard rank averagely in the top 17.44% with a standard deviation of 15.51%. This result indicates that highly likely SL interactions can be discovered through RRA analysis.

To verify the reliability of the results derived from the RRA analysis, we additionally compared it with the averaging ranking, where the percentile rankings based on all the evidence types are averaged with the same weights for each SL gene pair. Our results show that these golden standard SL pairs are ranked lower in terms of the percentile rankings derived from the averaging ranking method compared to that from the RRA method (Fig. S1), indicating that RRA is able to rank true SL pairs on the top of the list.

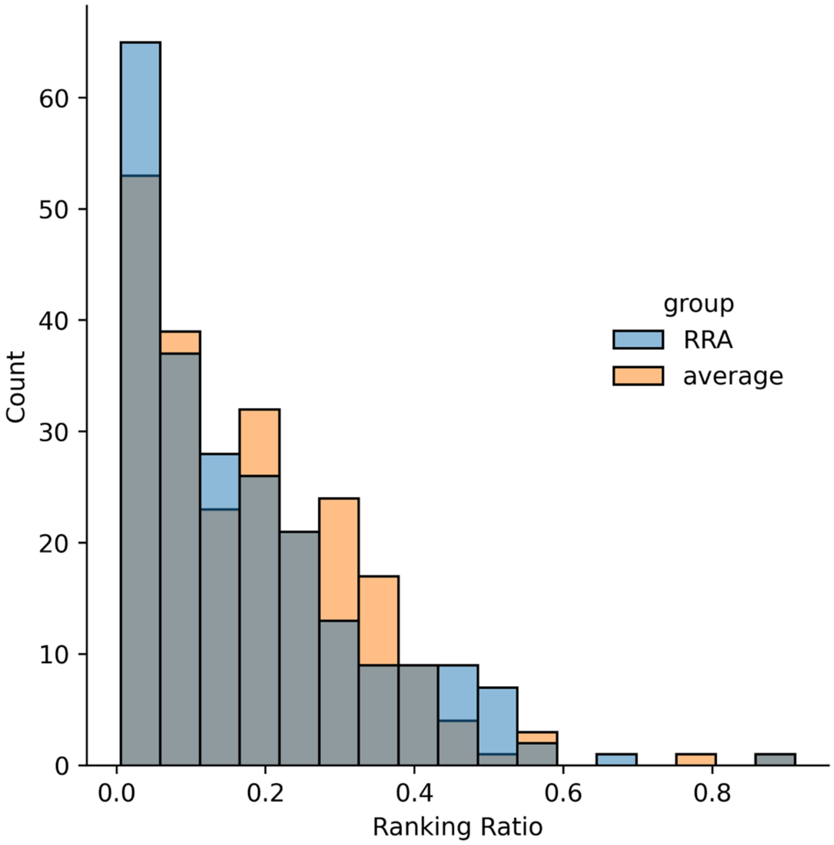

Figure S1: The distribution of the ranking ratios of golden partner gene among 176 SL gene pairs between RRA and average ranking method.

4 Case Studies

To illustrate the analysis process and interpretation of results by SL-Miner more comprehensively, we conducted two case studies as shown below. Our case studies show two examples of either using genes participating in the same pathway with the primary gene, or using paralog genes of the primary gene as the partner candidates, to mine SL evidence and prioritize the potential SL interactions.

4.1 SL interactions between BRCA1 and DDR-related genes

In the first case study, we used SL-Miner to prioritize potential SL interactions between BRCA1 and DNA damage response (DDR)-related genes in breast cancer.

A list of 333 DDR-related SL partner genes of BRCA1 was loaded from the database of MSigDB. Consequently, rank aggregation analysis was done in the Prioritization module. We selected breast cancer as input for TCGA and DepMap cancer type. The analysis was based on the ranking results of the screening evidence tests and omics evidence tests.

Table S1: Results of Rank Aggregation analysis of the top 30 DDR-related candidate SL partner genes of BRCA1 in breast cancer. Genes that can be found in SynLethDB as SL partners of BRCA1 are highlighted in red.

PTG Candidate	RRA Score	RRA Rank
MBD4	0.0009	1
POLE3	0.004	2
FEN1	0.0087	3
CHEK1	0.0144	4
RFC4	0.0158	5
TOPBP1	0.0158	6
RAD51D	0.0295	7
POLQ	0.03	8
PPP5C	0.0313	9
FANCA	0.0327	10
TIPIN	0.0356	11
RMI2	0.0371	12
FANCC	0.0372	13
EXO1	0.0387	14
POLE4	0.0409	15
RAD50	0.0453	16
ISY1	0.0472	17
RMI1	0.0472	18
USP1	0.0475	19
XRCC1	0.052	20
TIMELESS	0.052	21
ACTB	0.052	22
POLR2D	0.052	23
CDK2	0.0611	24
PARP2	0.0621	25
MSH2	0.0644	26
PARP1	0.0723	27
LIG3	0.0723	28
PCLAF	0.0744	29
RUVBL1	0.0771	30

The RRA rank scores showed that among the top 30 partner gene candidates, 10 of them are included in SynLethDB; among the top 10 candidates, 4 partner genes can be found in SynLethDB, which are FEN1, CHEK1, RFC4, and FANCA (Table S1). To verify whether the

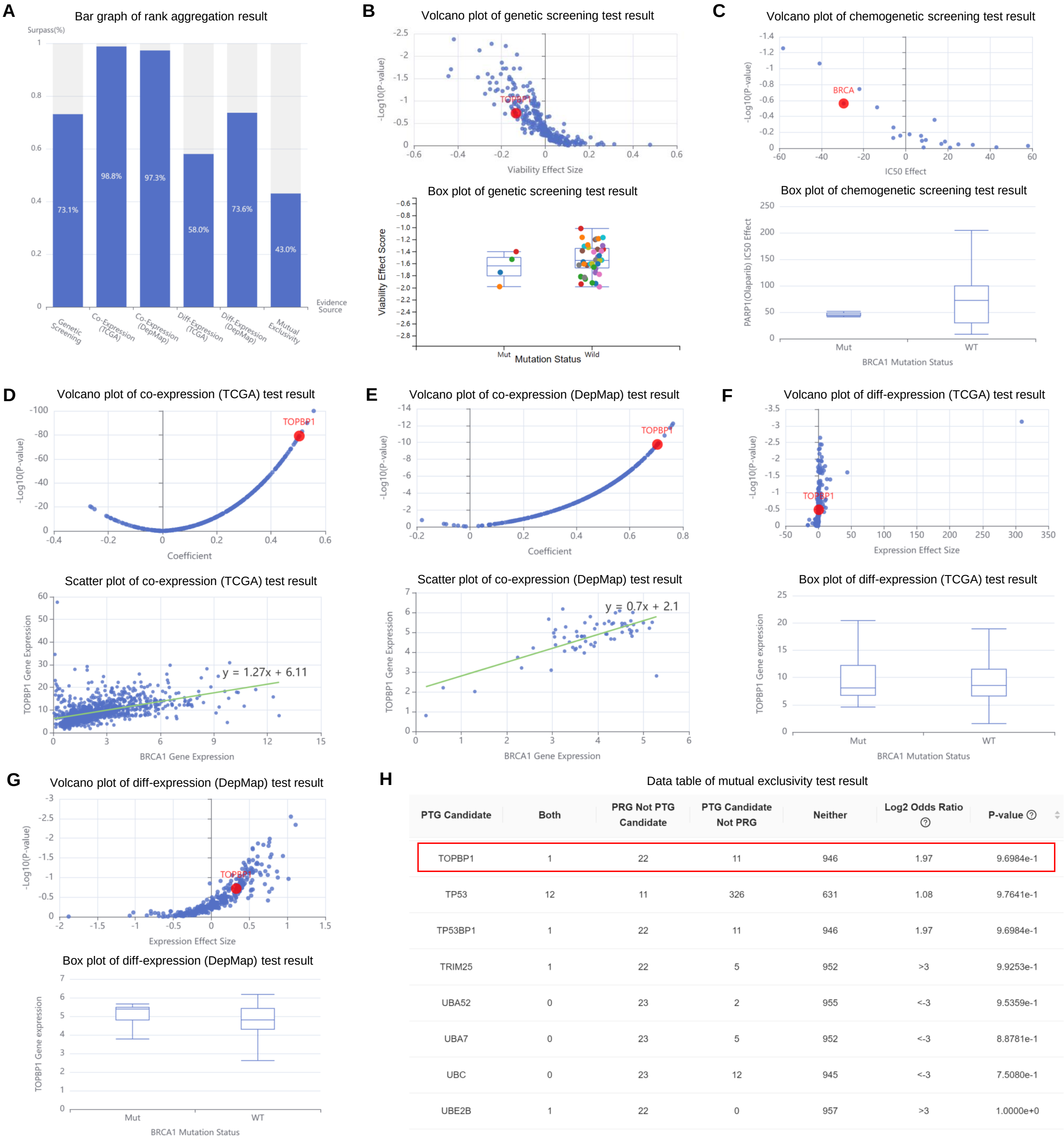

PTG Candidate	Both	PRG Not PTG Candidate	PTG Candidate Not PRG	Neither	Log2 Odds Ratio	P-value
TOPBP1	1	22	11	946	1.97	9.6984e-1
TP53	12	11	326	631	1.08	9.7641e-1
TP53BP1	1	22	11	946	1.97	9.6984e-1
TRIM25	1	22	5	952	>3	9.9253e-1
UBA52	0	23	2	955	<-3	9.5359e-1
UBA7	0	23	5	952	<-3	8.8781e-1
UBC	0	23	12	945	<-3	7.5080e-1
UBE2B	1	22	0	957	>3	1.0000e+0

Figure S2: The results of evidence mining of SL interactions between BRCA1 and DDR-related genes, focusing on TOPBP1 as the candidate SL partner gene. **(A)** The bar graph visualizes the ranking positions of TOPBP1 in terms of the 6 evidence tests included in the screening evidence and omics evidence tests. **(B)** The volcano plot displaying the $-\log_{10}$(P-value) and Viability Effect sizes of candidate partner genes from the DDR gene set, and the box plot visualizing the distribution of dependency scores of TOPBP1 between BRCA1-Mut group and WT group. **(C)** The volcano plot displaying the PARP1 inhibitor Olaparib's $-\log_{10}$(P-value) and IC50 scores, and the box plot visualizing the distribution of IC50 scores of Olaparib between BRCA1-Mut group and WT group. **(D)** and **(E)** The volcano plots displaying the $-\log_{10}$(P-value) and correlation coefficients of candidate partner genes from the DDR gene set, and the scatter plots showing gene expression correlation between TOPBP1 and BRCA1, on data sourced from TCGA and DepMap, respectively. **(F)** and **(G)** The volcano plots displaying $-\log_{10}$(P-value) and gene expression values of candidate partner genes from the DDR gene set and the box plots visualizing the distribution of expression effect sizes of TOPBP1 between BRCA1-Mut group and WT group, with data sourced from TCGA and DepMap, respectively. **(H)** Part of the data table displaying the 2×2 contingency table results as well as the $\log_2$ odds ratio and p-value derived from one-sided Fisher's exact test in the Mutual Exclusivity test. TOPBP1 is highlighted in the red box.

rest 6 partner genes (i.e. MBD4, POLE3, TOPBP1, RAD51D, POLQ and PPP5C) in the list of top ten could be novel SL partners of BRCA1, further evidence mining for each candidate is needed. Here we take TOPBP1 as an example. The bar plot shows that TOPBP1 ranks highly in terms of co-expression. In the Genetic Screening and Differential Gene Expression tests on the DepMap data, it ranks in the top 30%. While in the Differential Gene Expression and the Mutual Exclusivity tests on the TCGA data, it only ranks around the average position among all the candidates (Fig. S2A).

Data tables and visual plots of the results derived from each evidence test are accessible in the corresponding testing module. For instance, in the Genetic Screening test, an overview visualization of $-\log_{10}$(p-value) and the Viability Effect sizes of all the candidate partner genes is displayed as a volcano plot. Clicking on the "Inspect" button of TOPBP1 will highlight its position in the plot (Fig. S2B). Based on the hypothesis that for an SL gene pair the viability effect score of the partner gene will be significantly lower if its primary gene is mutated, and as such the gene pairs with lower viability effect sizes and lower p-values are more likely to have SL interactions. TOPBP1 is ranked moderately high with an effect size of -0.134 and a p-value of 0.190. The box plot displays the distribution of dependency scores of TOPBP1 between the BRCA1-Mut group and WT group (Fig. S2B). From the distribution, the viability effect scores of TOPBP1 in BRCA1-Mut samples are averagely lower than that in WT samples. However, since the Wilcoxon rank sum test derived p-value > 0.05, there is no significance for this result.

The results in other testing modules follow a similar procedure as described above. In the Chemogenetic Screening test, since no drug has yet been approved to target TOPBP1 in GDSC, we showed the case of PARP1 and BRCA1 instead solely for demonstration of the visualization (Fig. S2C). In the Gene Co-Expression test, TOPBP1 is significantly co-expressed with BRCA1. On TCGA, the Pearson correlation is 0.504 and the non-correlation test p-value is $8.51e^{-80}$ (Fig. S2D). On DepMap, the Pearson correlation is 0.704, and the p-value is $1.73e^{-10}$ (Fig. S2E). In the Differential Gene Expression test, TOPBP1's Expression Effect Size is 0.815 and the p-value derived from one-sided Wilcoxon rank sum test is 0.328 on TCGA (Fig. S2F). On DepMap, the Expression Effect Size is 0.328 and the p-value is 0.191 (Fig. S2G). In the Mutual Exclusivity test, the $\log_2$ odds ratio derived from the 2×2 contingency table is 1.97 and the p-value derived from the one-sided Fisher exact test is 0.970 (Fig. S2H).

4.2 SL interactions between paralog gene pairs

In the second case study, we used SL-Miner to prioritize potential SL interactions between paralog gene pairs, which are hereafter called paralog SL pairs.

The most promising paralog SL pairs were obtained from three combinatorial screening efforts, including 43 gene pairs in total. Among these paralog SL pairs, we selected those pairs including one gene with at least 10 paralogues. Paralogues for each gene were obtained from

Ensembl (Zerbino *et al.*, 2018). Moreover, we selected the cancer types as input according to the corresponding cell lines used in the screening experiments. We performed rank aggregation analysis to get the ranking positions of the paralog SL pairs we have selected (including ARFGEF1/ARFGEF2, CCNE2/CCNE1, CDK6/CDK4, CHD2/CHD1, CSNK1E/CSNK1D, SOS1/SOS2, HDAC1/HDAC2, MAPK3/MAPK1, OXSR1/STK39, SAR1A/SAR1B and TIAL1/TIA1). They rank in the top 24.14% on average, which indicates the ability of SL-Miner to find potential paralog SL pairs. The average aggregated ranking position of a gene pair was obtained by using the primary gene and their paralogues as input in the SL-Miner Prioritization module and then averaging the percentile rankings of the true partner gene across the cancer types (as input for context) of this gene pair.

Among the paralog SL pairs we have tested, we focused on CCNE1/CCNE2 in Colorectal cancer, which are both important in the regulation of the cell cycle. Following the similar analysis process in the first case study, we first observed the analysis results in the Prioritization module and then mined the supporting evidence in multiple evidence testing modules.

The bar plot in Fig. S3A shows that CCNE1 ranks highly in the Genetic Screening test. In the Differential Gene Expression test and the Mutual Exclusivity test, it ranks in the top 30%. In the Gene Co-Expression test, it surpasses 77.8% of other candidates on DepMap but only surpasses 22.2% of other candidates on TCGA (Fig. S3A). To find clues for the SL interaction between CCNE1/CCNE2, we further analyze the results in the evidence testing modules. In the Genetic Screening test, the $-\log_{10}$(p-value) of CCNE1 surpasses all the other candidates, showing that the cell lines with CCNE2 mutations are more sensitive to the deletion of CCNE1 than other cell lines (Fig. S3B). Co-expression patterns between CCNE1 and CCNE2 are moderate, especially on the TCGA data (with a Pearson correlation coefficient of 0.072 and p-value of 0.078 on TCGA, and a Pearson correlation coefficient of 0.356 and p-value of 0.002 on DepMap) (Fig. S3C,D). However, CCNE1 and CCNE2 do not show a strong tendency in mutual exclusive expression (with a p-value of 0.983) (Fig. S3G) or differential expression (with an expression effect size of 1.812 and p-value of 0.079 on TCGA, and expression effect size of 0.673 and p-value of 0.008 on DepMap) (Fig. S3E, F). The results are consistent with previous research showing that cyclins E1 and E2 (encoded by CCNE1 and CCNE2, respectively) are frequently expressed independently of one another in human cancer (Caldon and Musgrove, 2010). Supported by previous literature and the analysis results from SL-Miner, CCNE1/CCNE2 could be a promising paralog SL pair across multiple cancer types, although they do not have a strong expression correlation commonly seen in SL gene pairs (Jerby-Arnon *et al.*, 2014b).

In SL-Miner, all the data tables and data visualization plots generated during analysis can be downloaded in .csv (for tables) or .png (for plots) format directly.

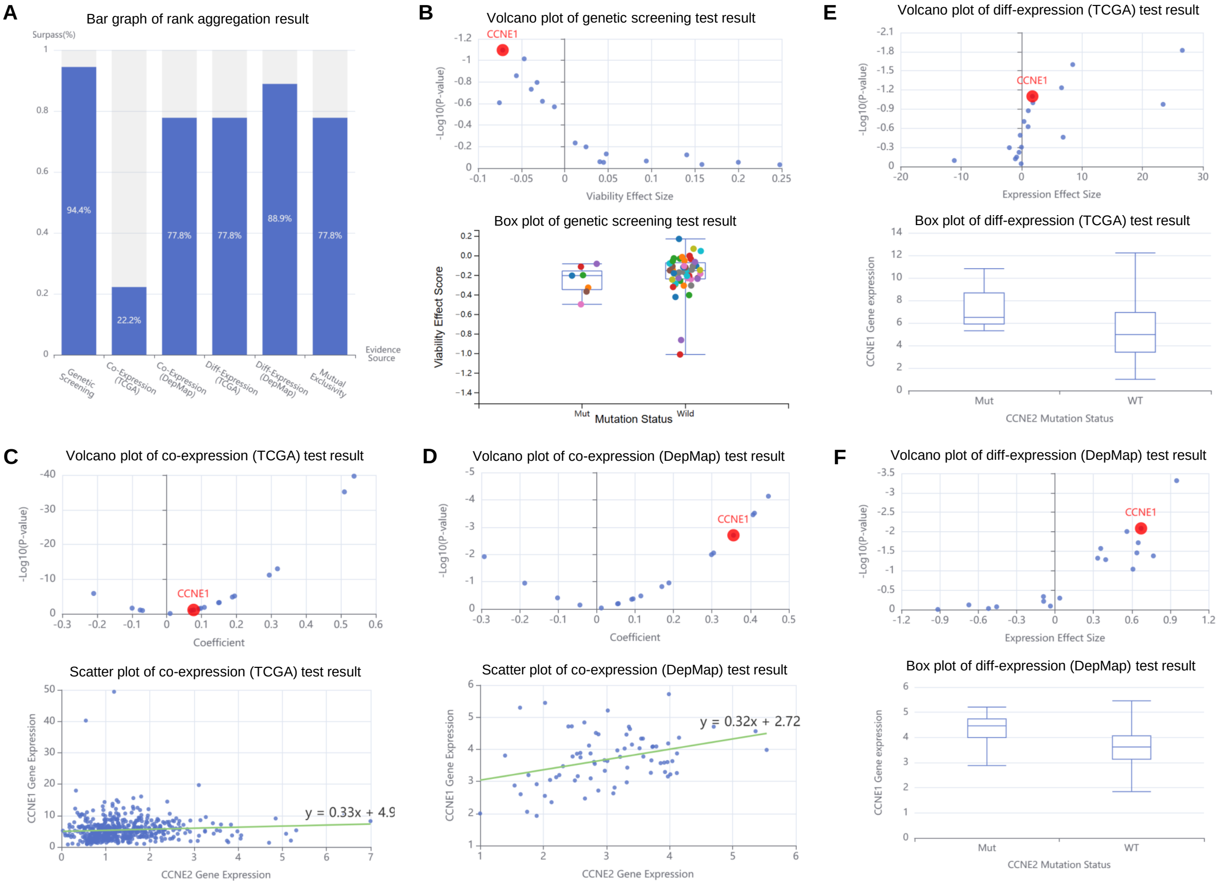

G Data table of mutual exclusivity test result

PTG Candidate	Both	PRG Not PTG Candidate	PTG Candidate Not PRG	Neither	Log2 Odds Ratio	P-value
CCNE1	0	3	5	431	<-3	9.6614e-1
CCND2	0	3	2	434	<-3	9.8636e-1
CCNF	1	2	8	428	>3	9.9889e-1
CCNP	0	3	1	435	<-3	9.9317e-1
CCNB1	0	3	7	429	<-3	9.5282e-1
CCND3	0	3	2	434	<-3	9.8636e-1

Figure S3: The results for example analysis of SL interactions between CCNE2 and CCNE2's paralogous genes, focusing on CCNE1 as the target candidate partner gene. **A** The bar graph visualizes the ranking positions of CCNE1 in terms of the 6 evidence tests included in the previous screening evidence and omics evidence tests. **B** The volcano plot displaying the $-\log_{10}$(P-value) and Viability Effect Size of each candidate partner gene from the DDR gene set, and the boxplot visualizing the distribution of dependency scores of CCNE1 between CCNE2-Mut group and WT group. **C** and **D** The volcano plot displaying the $-\log_{10}$(P-value) and correlation coefficient of each candidate partner gene from CCNE2's paralogous genes, and the Implot showing gene expression correlation between CCNE1 and CCNE2, data sourced from **C** TCGA and **D** DepMap, respectively. **E** and **F** The volcano plot displaying $-\log_{10}$(P-value) and gene expression of each candidate partner gene from CCNE2's paralogous genes and the boxplot visualizing the distribution of expression effect size of CCNE1 between CCNE2-Mut group and WT group, data sourced from **E** TCGA and **F** DepMap, respectively. **G** Part of the data table displaying the 2×2 contingency table results as well as the log2 odds ratio and p-value derived from one-sided Fisher's exact test in mutual exclusivity test. CCNE1 is highlighted within a red box.

5 References

Caldon, C. E. and Musgrove, E. A. (2010). Distinct and redundant functions of cyclin e1 and cyclin e2 in development and cancer. *Cell Division*, **5**, 1–13.

Dempster, J. M. *et al.* (2021). Chronos: a cell population dynamics model of CRISPR experiments that improves inference of gene fitness effects. *Genome Biol.*, **22**(1), 343.

Deng, Y. *et al.* (2019). Identifying mutual exclusivity across cancer genomes: computational approaches to discover genetic interaction and reveal tumor vulnerability. *Brief. Bioinform.*, **20**(1), 254–266.

Gallo, D. *et al.* (2022). CCNE1 amplification is synthetic lethal with PKMYT1 kinase inhibition. *Nature*, **604**(7907), 749–756.

Horlbeck, M. A. *et al.* (2018). Mapping the genetic landscape of human cells. *Cell*, **174**(4), 953–967.

Jerby-Arnon, L. *et al.* (2014a). Predicting cancer-specific vulnerability via data-driven detection of synthetic lethality. *Cell*, **158**(5), 1199–1209.

Jerby-Arnon, L. *et al.* (2014b). Predicting cancer-specific vulnerability via data-driven detection of synthetic lethality. *Cell*, **158**(5), 1199–1209.

Kanehisa, M. and Goto, S. (2000). KEGG: kyoto encyclopedia of genes and genomes. *Nucleic Acids Res.*, **28**(1), 27–30.

Kolde, R. *et al.* (2012). Robust rank aggregation for gene list integration and meta-analysis. *Bioinformatics*, **28**(4), 573–580.

Najm, F. J. *et al.* (2018). Orthologous crispr–cas9 enzymes for combinatorial genetic screens. *Nature Biotechnology*, **36**(2), 179–189.

Parameswaran, S. *et al.* (2019). A road map to personalizing targeted cancer therapies using synthetic lethality. *Trends in Cancer*, **5**(1), 11–29.

Shen, J. P. *et al.* (2017). Combinatorial crispr–cas9 screens for de novo mapping of genetic interactions. *Nature Methods*, **14**(6), 573–576.

Sinha, S. *et al.* (2017). Systematic discovery of mutation-specific synthetic lethals by mining pan-cancer human primary tumor data. *Nat. Commun.*, **8**, 15580.

Wang, J. *et al.* (2022). SynLethDB 2.0: a web-based knowledge graph database on synthetic lethality for novel anticancer drug discovery. *Database*, **2022**.

Zerbino, D. R. *et al.* (2018). Ensembl 2018. *Nucleic Acids Research*, **46**(D1), D754–D761.

Zhang, B. *et al.* (2021). The tumor therapy landscape of synthetic lethality. *Nat. Commun.*, **12**(1), 1275.
